# Circulating cell-free mitochondrial DNA as a candidate marker of hyperinflammation and immune activation in pre-adolescents, adolescents and young adults with COVID-19 and comorbidities

**DOI:** 10.1590/S1678-9946202668006

**Published:** 2026-01-30

**Authors:** Gabriella Bayler Novo, Emilly Henrique dos Santos, Karen Alessandra Rodrigues, Gabriel Acca Barreira, Mariana Okay Saippa, Maria Carolina Pires Cruz, Maria Fernanda Baduê Pereira, Heloisa Helena de Sousa Marques, Thelma Suely Okay

**Affiliations:** 1 Universidade de São Paulo, Faculdade de Medicina, Instituto de Medicina Tropical de São Paulo, Laboratório de Soroepidemiologia e Imunobiologia, São Paulo, São Paulo, Brazil; 2 Santa Casa de São Paulo, Faculdade de Ciências Médicas, São Paulo, São Paulo, Brazil; 3 Universidade de São Paulo, Faculdade de Medicina, Departamento de Pediatria, São Paulo, São Paulo, Brazil; 4 Faculdade Israelita de Ciências da Saúde Albert Einstein, São Paulo, São Paulo, Brazil; 5 Faculdade de Medicina do ABC, Santo André, São Paulo, Brazil

**Keywords:** COVID-19, DAMP, Adolescence, Comorbidities, Cell-free mitochondrial DNA, Biomarker

## Abstract

Youth encompasses pre-adolescence (10-14 years), adolescence (15-17 years), and young adulthood (18-24 years). Adolescents in general, particularly those with comorbidities, appear more susceptible to severe COVID-19, a vulnerability also observed in newborns and young infants. The mechanisms underlying this increased risk remain unclear, highlighting the need for early disease biomarkers. Circulating cell-free mitochondrial DNA (ccf-mtDNA), a damage-associated molecular pattern (DAMP), has been linked to systemic inflammation and immune activation during viral infections. This study evaluated plasma ccf-mtDNA levels in pre-adolescents, adolescents, and young adults with and without COVID-19, all presenting respiratory symptoms and predominantly harboring comorbidities, some with coinfections by other respiratory viruses. In this prospective study of 88 participants aged 12-21 years, half tested positive and half negative for SARS-CoV-2 by Reverse-Transcribed Polymerase Chain Reaction (RT-PCR). Comorbidities were present in 75% of COVID-19-positive and 54.5% of COVID-19-negative participants. Coinfections were detected in 52.3% and 25% of tested participants, respectively. Plasma ccf-mtDNA was quantified by a quantitative Real Time PCR (qPCR) targeting the mitochondrial NADH dehydrogenase 2 (ND2) gene or MT-ND2. COVID-19-positive participants exhibited significantly higher ccf-mtDNA levels than both symptomatic COVID-19-negative individuals and healthy controls (p<0.001). Although median levels were numerically higher in severe/critical compared with mild/moderate cases (7,769 vs. 4,649 ccf-mtDNA/mL), the difference was not statistically significant, likely due to limited sample size. In conclusion, elevated ccf-mtDNA distinguishes young individuals with COVID-19 and comorbidities from non-COVID-19 symptomatic participants and healthy controls. Although not linked to disease severity in this preliminary study, ccf-mtDNA may serve as an early biomarker of SARS-CoV-2-induced hyperinflammation and immune activation, supporting further targeted clinical investigations.

## INTRODUCTION

The global outbreak of Coronavirus Disease 2019 (COVID-19) caused by the severe acute respiratory syndrome coronavirus 2 (SARS-CoV-2) has posed an unprecedented challenge to public health systems worldwide^1^.

While the majority of patients, especially in the pediatric field, exhibit asymptomatic to mild-moderate infections, a minor proportion develop severe respiratory complications such as the acute respiratory distress syndrome (ARDS), eventually requiring intensive care and resulting in death^2^. However, the incidence of symptomatic disease among children has changed throughout the pandemic with the emergence of new SARS-CoV-2 variants such as Delta (B.1.617.2) and Omicron (B.1.1.529) leading to increased rates of children hospitalization^3,4^.

For reasons that are not well understood, hospitalization rates due to COVID-19 are higher among adolescents and infants under the age of one year compared to other pediatric age groups. During the period when the Delta variant predominated (second semester of 2021), 25% of children admitted to six US hospitals were aged less than one year, 17% were one to four years, 20% were five to 11 years, and 38% were aged 12 to 17 years^5^. In a recent review on clinical manifestations and outcomes of SARS-CoV-2 infections in pre-adolescents and adolescents (12-17 years), and in those aged less than six months, adolescence and young age have been considered risk factors for more severe COVID-19 along with obesity, diabetes, kidney diseases, pulmonary diseases excluding asthma, immunodepression conditions, and the presence of multiple comorbidities^6^.

This heterogeneous clinical course highlights the still necessary search for reliable biomarkers to identify pediatric patients at risk of severe outcomes early in the course of COVID-19^2^.

In this context, circulating cell-free mitochondrial DNA (ccf-mtDNA) has emerged as a promising biomarker of systemic inflammation and immune activation. When mitochondrial integrity is compromised by oxidative stress triggered by inflammatory processes or infections in general, ccf-mtDNA can be released into the cytoplasm and then to the extracellular environment acting as a potent damage-associated molecular pattern (DAMP). These mtDNA fragments have been shown to activate innate immune pathways, such as those of Toll-like receptor 9 (TLR9) and inflammasomes, leading to the amplification of inflammatory responses^7-9^.

In SARS-CoV-2 infection, mitochondrial dysfunction is implicated not only in the systemic inflammatory process initially termed Multisystem Inflammatory Syndrome in Children (MIS-C) associated with the cytokine storm. In 2021, the concept of Acute COVID-19-Associated Hyperinflammation was introduced in the 2^nd^ version of the American College of Rheumatology (ACR) clinical guideline for MIS-C hyperinflammatory state^10,11^.

The hyperinflammatory state is associated with viral replication and immune evasion. SARS-CoV-2 alters mitochondrial membranes physiology to form double-layer vesicles that favor viral RNA replication and simultaneously inhibiting the antiviral signaling via mitochondrial antiviral signaling proteins (MAVS). The process evolves to disruption of mitochondrial membranes and release of cf-mtDNA into the circulation in a way that the more mitochondrial DNA is released into the circulation, the more intense the systemic inflammatory activity will be^9^.

Elevated levels of circulating ccf-mtDNA have been associated with poor outcomes in a number of critical illnesses, including sepsis, trauma, and ARDS^12,13^. More recent studies have shown that in COVID-19, high plasma mtDNA levels correlate with increased disease severity, a higher probability of intensive care admission, need for intubation and ventilatory assistance, renal replacement therapy and increased mortality in adult patients^8^.

Given the growing evidence linking mitochondrial dysfunction to immune dysregulation and inflammation in COVID-19, the quantification of ccf-mtDNA using qPCR offers an opportunity to improve risk stratification, and better understand the pathophysiological mechanisms underlying severe disease in this special age group of young people^14^.

In this preliminary study, we aimed to determine whether ccf-mtDNA could distinguish pre-adolescents, adolescents and young adults with COVID-19, most of whom had comorbidities, from a non-COVID-19 group that also presented respiratory symptoms and predominantly had comorbidities, as well as from healthy young controls. We further investigated whether ccf-mtDNA levels were associated with disease severity.

## MATERIALS AND METHODS

### Ethics

This study was conducted as part of a main research project on pediatric COVID-19, approved by the Institutional Ethics Committee (CAAE Nº 30344420.6.0000.0008) on April 20, 2020. Written informed consent was obtained from parents or legal guardians, and assent was obtained from pre-adolescents and adolescents, in accordance with the main research protocol. A biorepository was also established to support future investigations. The specific study on ccf-mtDNA was approved as a satellite research project (CAAE Nº 73522723.0.0000.0068) on October 23, 2023.

### Study design and participants

This single-center, prospective laboratory study was conducted at a tertiary pediatric care institution using DNA samples obtained from a biorepository. The research took place between January 2024 and January 2025 as part of the first author's scientific initiation internship.

### Blood sample collection

Peripheral blood was obtained from all participants in the COVID-19 groups at presentation to the Emergency Department, corresponding to the acute phase of respiratory symptoms.

### Inclusion criteria

Participants in the COVID-19-positive group were required to have a positive RT-PCR test for SARS-CoV-2 in nasopharyngeal secretions (Allplex™ 2019-nCoV assay, Seegene Inc., Seoul, South Korea), while in the COVID-19-negative group they should test negative for SARS-CoV-2.

According to the World Health Organization, adolescence spans ages 10 to 19 years, 11 months, and 29 days^15-18^. However, in several countries, including the United States, individuals up to 21 years of age are still considered part of the pediatric population^18^. Based on this broader definition, participants aged 12 to 21 years were included in the study. Due to institutional restrictions, it was not possible to collect blood samples from healthy children for the negative control group. Therefore, we adopted the concept of "youth", which overlaps with adolescence and extends up to 24 years of age, encompassing three categories: pre-adolescents (10-14 years), adolescents (15-19 years), and young adults (20-24 years)^15-18^.

Therefore, COVID-19-positive and -negative groups comprised pre-adolescents (10-14 years) and adolescents/ young adults (15-21 years), matched 1:1 by sex and age, totaling 44 participants per group (25 females and 19 males). The negative control group (NC) consisted of 30 healthy young adults (15 males and 15 females) aged 18-24 years, who were blood bank donors and whose blood samples had been collected, processed and conveniently stored prior to the COVID-19 pandemic.

### Exclusion criteria

Participants were excluded if they had incomplete clinical data, any bacterium in blood, urine or other culture, insufficient DNA sample volume for analysis, or if their samples showed evidence of hemolysis or inadequate storage conditions. Participants whose parents did not consent, pre-adolescents or adolescents who did not assent in participating were also excluded.

### Type of comorbidity in the COVID-19 groups

Participants were categorized by the need of follow-up care in our institution in four categories: genetic diseases like inborn errors of immunity, cystic fibrosis, Rubinstein-Taybi syndrome, Down syndrome, Di George syndrome, systemic lupus erythematosus, among others; cancer (leukemia, lymphoma, nephroblastoma, central nervous system tumors, hepatocarcinoma, among others); serious malformations compromising neurodevelopment and feeding; and organ failure/ transplant (mainly respiratory, renal, or liver insufficiency)/ (liver, renal, or bone marrow transplantation).

### Pediatric COVID-19 severity

The severity of COVID-19 was classified between the sixth and eighth day after the participant's emergency care visit, as all participants presented with acute respiratory symptoms at that time, and the exact date of symptom onset could not be determined from the medical records. Participants were divided into four groups of disease severity (mild, moderate, severe and critical), as previously described^19^. In general, mild COVID-19 cases were followed-up in the outpatient clinic, moderate cases could be hospitalized according to their need of oxygen supplementation, while severe and critical cases were systematically hospitalized, and critical cases were admitted to the pediatric intensive care unit.

### Search for other 19 respiratory viruses

A commercial respiratory virus panel testing 19 other respiratory viruses (Luminex xTAG Respiratory Viral Panel FAST v2 assay (Luminex Molecular Diagnostics, Canada) was available. However, due to non-request by the attending physician or problems associated with work overload in the emergency room during the pandemic, the viral panel was only performed for 11 of 44 (25%) COVID-19-negative participants, and 23 of 44 participants (52.3%) in the COVID-19-positive ones. In the COVID-19-negative group, the 11 investigations resulted in 8 negative tests and three positive ones: rhinovirus only (n=1) and EBV (n=1) and one double identification coronavirus HKU1 + rhinovirus (n=1). In the COVID-19-positive participants, the 23 investigations resulted in 17 negative results and 6 positive ones: rhinovirus only (n=2), CMV (n=1), EBV (n=1), RSV (n=1), and one double identification (rhinovirus + parainfluenza 4).

### Cell-free mitochondrial DNA extraction from plasma samples

Whole blood samples were initially centrifuged to separate the plasma from the cells. Then, 200 uL of plasma underwent multiple centrifugation steps of 5 min at 800 g, followed by 15 min at 1,000 g in a refrigerated microcentrifuge to ensure a clear plasma sample free of cellular debris. Next, 200 uL of clear plasma were submitted to DNA extraction with the QIAamp DNA Mini Kit (QIAGEN, Hilden, Germany), following the manufacturer's protocol. Thereafter, DNA concentration was estimated in a Nanodrop 1000 spectrophotometer (Thermo Fisher Scientific, Waltham, Massachussets, USA).

### Quantification of circulating cell-free mitochondrial DNA (ccf-mtDNA) via quantitative PCR (qPCR)

Circulating cell-free mitochondrial DNA (ccf-mtDNA) in plasma was quantified by quantitative real-time PCR (qPCR) using the ABI StepOne Real-Time PCR System (Applied Biosystems, Thermo Fisher Scientific). The qPCR target gene was the mitochondrial NADH dehydrogenase 2 enzyme gene (MT-ND2). A fragment of 90 bp was amplified by the primers forward: 5′-CACAGAAGCTGCCATCAAGTA-3′ and reverse: 5′-CCGGAGAGTATATTGTTGAAGAG-3′ delineated along the MT-ND2 gene sequence.

### Cloned positive control for qPCR calibration curves

The 90 bp PCR product was cloned into a plasmid vector using the TA Cloning Kit (Invitrogen, Thermo Fisher Scientific). The total plasmid mass (vector + insert) was used to estimate copy number using the following formula, where X is the DNA mass and N is the total base pairs: Copies = (X ng × 6.022 × 10^23^) / (N × 660 × 10^9^). Then, serial dilutions of the cloned plasmid beginning with 10^6^ copies up to 1 copy/ microtube served as standard for the qPCR calibration curve to quantify ccf-mtDNA copy number in clinical samples.

### qPCR amplification protocol

Quantitative amplifications used the QuantiFast SYBR Green PCR Master Mix (QIAGEN), in a total volume of 25 uL containing 0.2 µM of both primers and 100 ng of template DNA. Cycling conditions were 95 °C for 10 min, then 40 cycles of 95 °C for 15 s and 60 °C for 60 s, ending with the melt curve. The qPCR calibration curve and clinical samples were tested in triplicate.

### Statistical analysis

The Shapiro-Wilk normality test for small samples (< 50) was applied. This test assesses if a sample's distribution comes from a normal population by comparing the sample data with a perfect normal (Gaussian) distribution. As the distribution of ccf-mtDNA results was not normal, non-parametric tests were employed, Mann-Whitney for comparisons between two groups, and Kruskal-Wallis with Dunn's post-hoc analysis and p-values adjusted by Bonferroni for comparison of three or more groups. To compare proportions between two groups the χ^2^ test or Fisher's exact test (when comparisons involved one or more subgroups with number of cases < 2) was used. Statistical analyses were performed with the GraphPad Prism, version 8.0 (GraphPad Software, Boston, MA, USA).

## RESULTS

In this study, a total of 44 pre-adolescents, adolescents and young adults with COVID-19 and 44 without COVID-19 matched by age and sex, in addition to a negative control group composed of 30 healthy young blood donors were evaluated.

Table 1 summarizes data from participants in the two COVID-19 groups (positive or negative) and in the negative control group, according to demographics, presence of coinfections with other respiratory viruses (RV), presence and type of comorbidity (CMB), and severity of COVID-19 when applicable.

**Table 1:** Distribution of participants according to COVID-19 status (positive or negative), demographic characteristics, presence of other respiratory viruses, comorbidities (including type), and disease severity when applicable.

Variables)	Participants N = 88		
	COVID-19 Positive N = 44	COVID-19 Negative N = 44	p-value
**Sex**			
Female	25 (56.8%)	25 (56.8%)	0.999
Male	19 (43.2%)	19 (43.2%)	
**Age group (years)**			
12-14	9 (20.4)	13 (29.5)	
15-19	18 (41.0)	22 (50.0)	0.170
20-21	17 (38.6)	9 (20.5)	
**Respiratory viruses**			
Detected	6 (13.7%)	3 (6.8%)	0.032
Undetected	17 (38.6%)	8 (18.2%)	
Not performed	21 (47.7%)	33 (75.0%)	
**Comorbidity**			
Yes	33 (75%)	24 (54.5%)	0.045
No	11 (25%)	20 (45.5%)	
**Type of comorbidity**	**N = 33**	**N = 24**	
Genetic diseases	18 (54.5%)	9 (37.5%)	0.420
Cancer	5 (15.2%)	4 (16.7%)	
Serious malformations	1 (3.0%)	3 (12.5%)	
Organ failure/ transplant	9 (27.3%)	8 (33.3%)	
**COVID-19 severity**	**N = 44**		
Mild	23 (52.3%)	NA	NA
Moderate	7 (15.9%)	NA	
Severe	7 (15.9%)	NA	
Critical	7 (15.9%)	NA	

Chi-square (c^2^) test or Fisher's exact test compared proportions between groups. COVID-19 severity was classified into four groups (mild, moderate, severe and critical) according to Dong *et al*.^20^; NA = not applicable.

The Shapiro-Wilk normality test was applied and showed that ccf-mtDNA data did not follow a normal distribution. Median values of ccf-mt-DNA in copies/ mL in the three groups (CI 95%, min.-max. values) were:

– COVID-19 positive group: median 5,447 copies/mL (95% CI, min.184.2 -max. 185,832);
– COVID-19 negative group: 1,955 (95% CI, min. 104.8 - max. 20,588);
– Negative control group: 486.7 (95% CI, min. 32.37 - max. 2,811).

Table 2 shows the descriptive statistical analysis of circulating cell-free mitochondrial DNA (ccf-mtDNA, expressed in copies/mL) across the three study groups: COVID-19-positive, COVID-19-negative, and negative controls.

**Table 2:** Descriptive statistical analysis of circulating cell-free mitochondrial DNA (ccf-mtDNA), expressed in copies/ mL across the three study groups: COVID-19-positive, COVID-19-negative, and negative controls.

	Negative Control (NC)	COVID-19 positive	COVID-19 negative
Number of values	30	44	44
Minimum value of ccf-mtDNA (copies/mL)	32.37	184.2	104.8
25% Percentile	296.4	2,281	873.8
Median value of ccf-mtDNA	486.7	5,447	1,955
75% percentile	717.1	12,988	3,613
Maximum value of ccf-mtDNA	2,811	185,832	20,588
Range	2,778	185,648	20,483
Mean	649.4	13,585	3,134
Srandard deviation	660.7	28,737	4,209
Standard error of mean	120.6	4,332	634.5
Coefficient of variation of ccf-mtDNA	101.7%	211.5%	134.3%

Then, the non-parametric Kruskal-Wallis test compared ccf-mtDNA levels in the three groups, resulting in a highly significant statistical difference (p-value < 0.0001). Thereafter, to determine which of the pairwise comparisons yielded the significant difference, Dunn's post-hoc test with p-values adjusted by Bonferroni was applied revealing that the three pair-wise comparisons generated statistically significant differences (COVID-19-positive vs. NC; COVID-19-negative vs NC; COVID-19-positive vs. COVID-19-negative) (Table 3).

**Table 3:** Dunn's post hoc test, following the Kruskal-Wallis analysis for pairwise group comparisons.

Dunn's multiple comparisons test	Mean rank diff.	Significant?	Summary	Adjusted p-value
NC vs. COVID-19-positive	-55,71	Yes	****	<0.0001
NC vs. COVID-19-negative	-29,75	Yes	***	0.0007
COVID-19-positive vs. -negative	25,95	Yes	**	0.0011

Thereafter, Table 4 shows the descriptive statistical analysis of ccf-mtDNA levels from the COVID-19-positive cases divided into two groups according to disease severity (mild and moderate cases vs. severe and critical ones). It is noticeable that the mean ccf-mtDNA levels (copies/mL) in the two groups are quite different (10,059 vs. 21,139) as severe and critical cases have more than double the number of copies/mL of ccf-mtDNA with respect to the group of mild and moderate cases. At the same time, median values were 4,649 and 7,769 copies/mL, respectively, also pointing to differences of ccf-mtDNA levels between the two subgroups of disease severity.

**Table 4:** Descriptive analysis of ccf-mtDNA levels (copies/mL) in the COVID-19-positive group, subdivided into mild/moderate and severe/critical cases.

	Mild/moderate COVID-19	Severe/critical COVID-19
Number of ccf-mtDNA values in each severity group	30	14
Minimum value	184.2	1,028
25% percentile	2,056	2,381
**Median of ccf-mtDNA values**	**4,649**	**7,769**
75% percentile	13,180	14,887
Maximum	42,030	185,832
Range	41,846	184,804
**Mean of ccf-mtDNA values**	**10,059**	**21,139**
Standard deviation	12,563	47,847
Lower 95% CI of mean	5,368	-6,487
Upper 95% CI of mean	14,751	48,765
Standard error of mean	2,294	12,788
Coefficient of variation of ccf-mtDNA values	125%	226%

In the COVID-19-positive group there were 30 mild/ moderate cases, and 14 severe/ critical cases. When the median values of ccf-mtDNA in these two groups (4,649 × 7,769 ccf-mtDNA/mL) were compared by the Mann-Whitney test, the result did not reach statistical significance (p-value of 0.389) even though they were numerically different.

Then, we used Fisher's exact test to compare COVID-19-positive cases with and without coinfection by other respiratory viruses (Table 5), and no statistical difference was found (p= 0.530), bearing in mind that the subset of participants in both groups who underwent this investigation was very small. Consequently, in the group of severe and critical cases, the statistical test was not applied because one of the categories had n < 2.

**Table 5:** Comparison of median ccf-mtDNA levels (copies/mL) in COVID-19-positive participants according to the presence of respiratory virus (RV) coinfection and disease severity.

COVID-19 positive	With RV (N=6)	Without RV (N=17)	p^-^value
ccf-mtDNA (mild + moderate)	5 cases (11,684 copies/mL)	7 cases (13,186 copies/mL)	0.530
ccf-mtDNA (severe + critical)	1 case (20,582 copies/mL)	10 cases (25,860 copies/mL)	Not calculated

The Fisher exact test was applied when n>2 in subgroups; ccf-mtDNA - circulating cell-free mitochondrial DNA is given in copies/mL; RV = respiratory viruses.

The following analysis also used Fisher's exact test to compare the ccf-mtDNA levels of participants with or without comorbidities (CMB) and according to COVID-19 severity. No statistical differences were found, as the p-value was 0.504 in the severity group of mild to moderate COVID-19, and of 0.923 in the severe and critical group (Table 6).

**Table 6:** Comparison of median ccf-mtDNA levels (copies/mL) in COVID-19-positive participants according to the presence of comorbidities (CMB) and disease severity.

COVID-19 positive	With CMB (N=33)	Without CMB (N=11)	p^-^value
ccf-mtDNA (mild+ moderate)	21 (9,827 copies/mL)	9 (10,602 copies/mL)	0.504
ccf-mtDNA (severe + critical)	12 (23,394 copies/mL)	2 (7,609 copies/mL)	0.923

The Fisher exact test was applied as n> 2; ccf-mtDNA -circulating cell-free mitochondrial DNA is given in copies/mL; CMB = comorbidity.

## DISCUSSION

Adolescence represents a critical period of immune system maturation and heightened reactivity driven by hormonal changes during puberty. These hormonal shifts enhance adaptive immune functions, including cytokine signaling and antibody production, thereby improving protection against infections^20-25^. However, this increased immune activity also coincides with a three-fold higher susceptibility to autoimmune diseases, in which immune responses mistakenly target self-antigens^22,26,27^. Moreover, the adolescent immune system exhibits increased sensitivity to social and psychological stress, which can amplify inflammatory and even immune responses to social challenges^28,29^.

In this study, we found that circulating cell-free mitochondrial DNA (ccf-mtDNA) levels were markedly elevated in pre-adolescents, adolescents, and young adults with comorbidities who had confirmed COVID-19, compared with both symptomatic COVID-19-negative participants and healthy young controls (Table 3).

Table 1 showed that the two COVID-19 groups were comparable in terms of age, sex, and type of comorbidity, although slightly more participants in the COVID-19-positive group had comorbidities (p = 0.045). Given the very low number of participants tested for other respiratory viruses in both COVID-19 groups, the statistical difference observed in this comparison should not be considered.

Furthermore, Tables 5 and 6 found no differences in the presence of other respiratory viruses or comorbidities within the COVID-19-positive group when these participants were stratified by disease severity (mild/moderate vs. severe/critical). These non-significant comparisons should also be interpreted with caution, as the small number of cases required the use of Fisher's exact test.

Chronic pediatric conditions, including genetic, neurological, and autoimmune disorders, can impair mitochondrial metabolism and promote persistent inflammation, which may explain a mild baseline increase in ccf-mtDNA already observed even in the absence of acute infection. Children with such comorbidities often exhibit chronic low-grade or "silent" inflammation, a persistent, subclinical immune activation that gradually contributes to tissue damage and organ dysfunction. Unlike acute inflammatory or infectious states, silent inflammation does not produce overt clinical symptoms, but represents a fundamental pathophysiological mechanism underlying comorbidities like chronic kidney disease, diabetes, cardiovascular disease, and hypertension^30-33^.

In our cohort, although a pre-pandemic baseline evaluation was not available, it is plausible that pre-adolescents, adolescents, and young adults with comorbidities already exhibited mildly elevated ccf-mtDNA levels, irrespective of SARS-CoV-2 infection, indicating pre-existing mitochondrial stress and immune activation, as discussed in the previous paragraph^20-24^ COVID-19, however, seems to have further amplified this response, leading to a more pronounced release of ccf-mtDNA, particularly among those with comorbidities^34^. The significantly higher ccf-mtDNA levels observed in COVID-19-positive participants suggest that mtDNA release was primarily associated with SARS-CoV-2 infection rather than general respiratory illness. Most participants in the COVID-19-negative group also had comorbidities and showed moderate ccf-mtDNA elevations compared with both COVID-19-positive patients and healthy controls. Nevertheless, this increase was less pronounced and statistically weaker, consistent with milder mitochondrial disruption and less intense systemic inflammation and immune activation in this group (Table 3).

Elevated ccf-mtDNA has been associated with disease severity in adults, including ICU admission, need for ventilatory or renal support, and mortality^8,34^. In pediatrics, higher mtDNA levels have also been linked to oxygen supplementation requirements, supporting their potential inclusion in pediatric early warning scores (PEWS)^35^. Our findings extend these observations to pre-adolescence, late adolescence and early adulthood, suggesting that similar mechanisms of mitochondrial injury and hyperinflammation operate in this population, particularly in those with comorbidities.

While most pediatric SARS-CoV-2 infections are asymptomatic or mild, adolescents and infants under one year have consistently shown higher hospitalization and severe disease rates, especially during Delta and Omicron variant surges^3-6^. Epidemiological data indicate that adolescents constitute a significant proportion of hospitalized pediatric COVID-19 cases, particularly when comorbidities such as obesity, diabetes, chronic kidney or pulmonary disease, or immunosuppression are present^35^. These data motivated the focus on pre-adolescence, adolescence and early adulthood in our study, and highlighted the need for timely diagnostic and prognostic biomarkers in this high-risk population.

Mechanistically, SARS-CoV-2 has been shown to disrupt mitochondrial dynamics, promoting the release of mitochondrial contents and contributing to hyperinflammation and immunopathology. Extracellular mtDNA fragments act as damage-associated molecular patterns (DAMPs), activating TLR9 and inflammasome pathways, and fueling cytokine production and multi-organ dysfunction^9-12^.

Our findings suggest that similar mechanisms may underlie the elevated ccf-mtDNA observed in pre-adolescents, adolescents, and young adults with COVID-19 and comorbidities, although these mechanisms may be modulated by their generally more mature and, at times, more robust immune responses compared with those of newborns and older adults^21,23,24^.

Notably, while conventional inflammatory biomarkers such as C-Reactive Protein (CRP), Interleukin-6 (IL-6), and procalcitonin (PCT) are widely used in adults, their predictive value in pediatric populations depend on the clinical setting^36^.

In this research, we shared an institucional biorepository of pediatric patients with COVID-19. However, as many studies were conducted simultaneously, several of them on inflammatory biomarkers such as routine CRP, as well as pro-inflammatory cytokines (IL-6 and others), we were unable to expand our investigation to other inflammatory biomarkers. Nevertheless, our institution's data on inflammatory biomarkers in this specific scenario of pediatric COVID-19 have been released^37-39^.

Our findings support the utility of ccf-mtDNA as a more specific indicator of hyperinflammation in pre-adolescents, adolescents and young adults with COVID-19 and comorbidities. Quantification by qPCR is practical and scalable, allowing for serial monitoring from the acute phase through recovery, and may help identify patients at higher risk of severe disease or complications. This approach could also facilitate integration of ccf-mtDNA measurement into routine clinical workflows.

Although higher levels of ccf-mtDNA were observed when mean and median ccf-mtDNA levels of severe and critical COVID-19 cases were compared with mild and moderate ones (Table 4), this difference did not reach statistical significance, probably reflecting the small number of severe cases in our sample (n=14), the wide variability in ccf-mtDNA levels, or confounding factors that were not controlled such as the type of comordidity and the timing of sample collection, as patients presented to the hospital at varying times after respiratory symptoms onset, sometimes only after realizing their illness was more than a common cold. It is worth noting that all participants were sampled upon arrival at the Emergency Department, but this does not ensure they were at the same stage of disease progression. Similar findings have been reported in smaller adult cohorts, whereas larger studies have demonstrated significant associations with ICU admission and mortality^8,11-13,35^. It is also possible that ccf-mtDNA levels in pediatric patients plateau at a certain threshold or that pre-adolescents, adolescents and young adults regulate mitochondrial damage differently from older adults.

The high variability in ccf-mtDNA, particularly among COVID-19-positive participants, likely reflects heterogeneous host responses, influenced by individual immune and inflammatory profiles, timing of sample collection relative to symptom onset, and presence of underlying health conditions^34,35^.

This study has some strengths, including its prospective design, age- and sex-matched COVID-19-positive and -negative groups, and inclusion of healthy young controls. The use of non-parametric statistical tests ensured robustness despite undeniable sample size limitations. However, limitations should be acknowledged: the overall sample size was modest, especially regarding severe cases; the cross-sectional design precluded longitudinal assessment (serial monitoring from the acute phase through recovery or adverse outcome); comprehensive testing for viral co-infections was very limited as they have rarely been requested; medication effects, particularly of immunosuppressants were not evaluated; and precise timing of symptom onset relative to sample collection was not consistently documented.

## CONCLUSION

The results of this preliminar study highlight the potential of ccf-mtDNA as a laboratory biomarker for early identification of pre-adolescents, adolescents and young adults mostly with comorbidities at increased risk of hyperinflammation and clinical deterioration due to COVID-19. Elevated ccf-mtDNA appears to reflect both underlying comorbidities and SARS-CoV-2-related mitochondrial stress, offering a mechanistic link between infection, immune and inflammatory activation, and disease severity.

Future studies should include larger, multicenter cohorts with longitudinal sampling, stratification by comorbidity type and medications used, and a comprehensive assessment of viral coinfections to fully evaluate the prognostic value of ccf-mtDNA. Such investigations could pave the way for the integration of ccf-mtDNA into pediatric COVID-19 risk assessment and management protocols, ultimately improving early intervention strategies for high-risk pre-adolescents, adolescents and young adults.

## Data Availability

The complete anonymized dataset supporting the findings of this study is included within the article itself.
